# Ayurveda Management of Allergic Rhinitis: Protocol for a Randomized Controlled Trial

**DOI:** 10.2196/56063

**Published:** 2024-09-25

**Authors:** Shweta Mata, Shivshankar Rajput, Isha Preet Tuli, Pallavi Mundada, Bharti Gupta, Narayanam Srikanth, Rabinarayanan Acharya

**Affiliations:** 1 Central Ayurveda Research Institute Central Council for Research in Ayurvedic Sciences Ministry of Ayush New Delhi India; 2 Vardhman Mahavir Medical College Safdarjung Hospital New Delhi India; 3 Central Council for Research in Ayurvedic Sciences Ministry of Ayush New Delhi India

**Keywords:** allergic rhinitis, AR, Anu Taila Nasya, fluticasone propionate nasal spray, Naradiya Laxmivilas Rasa, randomized controlled trial, Shrishadi Kwath

## Abstract

**Background:**

Allergic rhinitis (AR) is the inflammation of the membranes lining the nose due to allergen exposure and is characterized by sneezing, nasal congestion, itching of the nose, or postnasal discharge. The prevalence varies worldwide, perhaps due to the geographic and aeroallergen differences, with 10% to 30% of the world’s population experiencing AR. In this study, Anu Taila Nasya, Naradiya Laxmivilas Rasa, and Shirishadi Kwath will be compared to a fluticasone nasal spray.

**Objective:**

The primary aim is to assess the efficacy of Ayurvedic management for AR (or vataja pratishyaya) by comparing it to a conventional control group. The secondary aims are to determine the mean change in the nasal endoscopy index and the mean change in the laboratory tests.

**Methods:**

This ongoing study is an open-label randomized controlled interventional trial, with a sample size of 90 both in the trial and standard control group (including dropouts, 20%), and will be carried out for 24 months. Participants in the trial group will receive Ayurvedic treatment, that is, Anu Taila Nasya (6 drops in each nostril for 7 days for 3 consecutive weeks), Naradiya Laxmivilas Rasa (250 mg twice per day), and Shirishadi Kwath (40 ml twice per day for 45 days). The participants in the control group will receive a fluticasone propionate nasal spray (2 sprays once per day for 45 days). The primary outcome will include the mean change in the Control of Allergic Rhinitis and Asthma Test score, and the secondary outcomes will include the mean change in the nasal endoscopy index (assessment of nasal membrane color, pale or hyperemia; rhinorrhea, watery or yellow; and inferior turbinate swelling, hypertrophy) and the mean change in the laboratory tests.

**Results:**

As of May 2024, 72 patients have been enrolled in both groups. Data analysis should be completed by February 2025. The study will be reported following standard guidelines for reporting randomized controlled trials. Clinical results will be disseminated through conferences and peer-reviewed publication in a relevant journal.

**Conclusions:**

The Ayurvedic approach could be an evidence-based therapeutic tactic for the management of AR.

**Trial Registration:**

Clinical Trials Registry India CTRI/2023/06/053395; https://tinyurl.com/564d2zz8

**International Registered Report Identifier (IRRID):**

DERR1-10.2196/56063

## Introduction

Allergic rhinitis (AR), a heterogeneous disorder, often remains undiagnosed despite its high prevalence. It is characterized by one or more of the following symptoms: sneezing, itching, nasal congestion, and rhinorrhea [1]. The nose is exposed to a variety of microorganisms, allergens, and environmental pollutants present in the atmosphere because of its direct contact with the external environment. If the early stage of AR is not adequately treated, it causes mucosal changes in nostrils and results in turbinate hypertrophy, nasal polyps, allergic bronchitis, etc. AR is the inflammation of the membranes lining the nose due to an allergen exposure [2].

AR affects 26% of the population in India [3]. The prevalence of AR has increased significantly in some countries, but national trends are inconsistent [4]. AR is the most common atopic condition, affecting about 10%-30% of adults and up to 40% of children worldwide [5]. In India, its prevalence has gradually risen in the last 2 decades [6]. The diagnosis is based on clinical manifestations and supported by positive results from a skin prick test or demonstration of serum-specific immunoglobulin E antibodies to aeroallergens or direct nasal endoscopy.

In this study, the diagnosis of AR and its classification into mild and moderate-severe groups were made according to the Allergic Rhinitis and Its Impact on Asthma (ARIA) classification [7]. The cardinal symptoms, according to ARIA guidelines, are paroxysmal sneezing; itching in the nose; itching of the eyes, palate, or pharynx; watery nasal discharge; nasal obstruction; and history of urticaria, while the clinical signs include pale boggy nasal mucosa; hypertrophied turbinates; thin, watery, or mucoid discharge; allergic shiner; and transverse nasal crease (allergic salute) [8]. The control group was selected as per ARIA guidelines.

The lack of curative treatment necessitates other systems of health providing remedial treatment, such as Ayurvedic science. As treatments with standard medications have their own limitations, there is scope for using Ayurveda, which emphasizes a holistic approach to every disease based on the Doshas (psychobiological rhythm: vata, pitta, and kapha), Dhatus (body tissues and their nourishing elements), and malas (excreta). In Ayurveda, AR is similar to vataja pratishyaya [9], and as per the Ayurvedic literature, it is a curable disease [10]. There are few published preclinical and clinical studies proving the efficacy of Ayurveda interventions, such as Rajanyadi Churna and Guduchi Kwath [11], Ayurvedic herbo-mineral formulation IMMBO [12], Triphala [13], Haridra Khanda and Manjisthadi Kwath (brihat) [14], Tamalakyadi decoction [15], and Haridra Khanda and Pippalyadi Taila [16].

One study was carried out at the Institute of Post Graduate Teaching and Research in Ayurveda, Jamnagar [17], and one pilot study was also conducted at the Central Council for Research in Ayurvedic Sciences (CCRAS) studying an Ashwagandhadi compound, Shirishadi Kwath, and Anu Taila Nasya (intranasal drug administration), which found significant results [18]. Naradiya Laxmivilas Rasa’s (NLR) use for respiratory disorders has previously been discussed in Ayurvedic literature [19]. No randomized controlled trial studies have been published comparing NLR, Shirishadi Kwath, and Anu Taila Nasya to a fluticasone nasal spray used in an allopathic system as per standard treatment for patients with AR. Considering these facts, a randomized controlled trial has been proposed here.

## Methods

### Study Objectives

The primary objective is to assess the efficacy of Ayurvedic management for AR (vataja pratishyaya) by comparing it with a conventional control group. The secondary objectives are to determine the mean change in the nasal endoscopy index (assessment of nasal membrane color, pale or hyperemia; rhinorrhea, watery or yellow; and inferior turbinate swelling, hypertrophy) [20] and mean change in the laboratory tests.

### Study Setting

This study will be conducted at Vardhman Mahavir Medical College and Safdarjung Hospital, New Delhi, India.

### Eligibility Criteria

#### Inclusion Criteria

Patients of either sex between the ages of 18 and 65 years with intermittent or persistent AR (as per the internationally recognized ARIA diagnostic standard) who were diagnosed based on their medical history and who were willing to participate and provide informed consent were eligible for the study.

#### Exclusion Criteria

The following exclusion criteria were used: patients who had a history of chronic nasal or upper respiratory tract symptoms or disorders other than AR, non-AR chronic sinusitis, or serve bronchial asthma with nasal anatomical defects (grossly deviated nasal septum), nasal polyposis, tonsillitis, or airway obstructions (adenoids); patients with a nasal condition likely to affect the outcome of the study; patients receiving any treatment of H1 antihistamine medication or nonsteroidal analgesics; patients using corticosteroids nasal drops, leukotriene antagonists, or nasal vasoconstrictors; patients who have received a surgical procedure on the nose like septoplasty/submucous resection, inferior turbinate reduction, or endoscopic sinus surgery; women who are pregnant and lactating mothers; patients with any debilitating chronic disease (eg, tuberculosis or uncontrolled diabetes mellitus), any unstable cardiovascular diseases, evidence of malignancy, concurrent hepatic disorder (defined as aspartate amino transferase or alanine amino transferase >2 times the upper normal limit) or renal disorders (defined as *S creatinine* greater than the upper limit of laboratory value), pulmonary dysfunction (bronchial asthma or chronic obstructive pulmonary disease), or acute mental disorder; and patients who are currently participating in any other clinical trial.

### Study Interventions (Investigational Products)

Participants in the treatment group will undergo Nasya therapy for 7 days for 3 consecutive cycles. Along with the Nasya therapy, the participants will receive a 250 mg dose of NLR twice daily after meals with lukewarm water and 40 ml of Shirishadi Kwath twice daily on an empty stomach for 45 days. In the standard control group, participants will receive a 200 mcg dose of fluticasone propionate nasal spray (50 mcg/spray) per day (2 puffs once daily) for 45 days. The treatment group medicine (investigational products) will be procured from good manufacturing practice–certified pharmacies through the Indian Medicines Pharmaceutical Corporation Limited (Mohaan) and the Central Ayurveda Research Institute (Jhansi), along with a certificate of analysis to ensure the investigational products’ quality standards and safety. The control group’s medicine will be procured from a good manufacturing practice–certified government agency. The study drugs will be kept in a secure place and will only be supplied to the participants under the guidance of the investigator. A record will be maintained for the drug dispensed. Any discrepancies between the amounts dispensed and returned will be explained.

### Strategies to Improve Adherence to the Study Protocol

Medication adherence will be assessed at each follow-up visit (days 16, 31, 45, 75, 105, and 135) by assessing the approximate quantity consumed. The participant will be asked to return the empty medicine container at each follow-up visit. Repeated phone reminders to regularly take the medicine from project staff will be given to the participants or their family members.

### Outcome Measures

#### Primary Outcome Measure

The primary outcome measure is the mean change in the Control of Allergic Rhinitis and Asthma Test (CARAT) score [21] at baseline and days 16, 31, 45, 75, 105, and 135. The CARAT questionnaire comprises 10 questions about upper and lower airway symptoms, sleep interference, activity limitation, and the need to increase medication over a 4-week period (Multimedia Appendix 1, also available on the web [22]). The total possible score ranges from 0 (minimum control) to 30 (maximum control) [21].

#### Secondary Outcome Measures

The secondary outcome measures are the mean change in the nasal endoscopy index (assessment of nasal membrane color, pale or hyperemia; rhinorrhea, watery or yellow; and inferior turbinate swelling, hypertrophy) and the mean change in laboratory tests at baseline and on day 45.

The nasal endoscopy index will be evaluated using the assessment method by Kim et al [20]. The doctor assesses the nasal membrane and inferior turbinate of the patient via nasal endoscopy. When evaluating the nasal membrane color, either the score for paleness or hyperemia must be 0 (eg, if the pale score is 2, the hyperemia score should be 0; if the nasal membrane color is normal, both pale and hyperemia scores are 0). The same rule is applied in the case of the rhinorrhea score. If there is no rhinorrhea, the scores for wateriness and yellowness are 0, and the yellow score is measured by evaluating the rhinorrhea parameter. An inferior turbinate swelling score is used to evaluate the nasal obstruction (Multimedia Appendix 2).

### Timelines With Deliverables

The total study period is 24 months and divided into 3 cycles (ie, 1-3.5 months for the treatment and standard trial drugs) along with the procurement of the certificate of analysis and finalization of the laboratory tests. Staff recruitment, purchases of equipment and other logistics, finalization of case report forms (CRFs), etc, were also done during this period. The timeline for participant recruitment will be 15 months, the treatment period will be 1.5 months, and the follow-up period will be 3 months. Data compilation, analysis, report preparation, and publication will be done during the last month.

### Sample Size

In a previous pilot study on this Ayurvedic intervention, a change of 14 points in the CARAT score was observed. For this study, it was assumed that a change of 10 points would be seen in the placebo group. With an SD of 6 points based on the results of the previous pilot study, a sample size of 35 participants per group was calculated to provide 80% power, with a 95% CI.

Assuming a dropout rate of 20%, a total of 90 participants, 45 per group, will be included in this trial.

n_1_ = n_2_ = 2 *σ*^2^ [(Z_1 – α/2_) + (Z_β_)]^2^ ÷ Δ^2^

n_1_ = n_2_ = 2 * (6)^2^ [(1.96 + 0.84)]^2^ ÷ (4)^2^

n_1_ = n_2_ = 35

where Δ is |μ_2_ – μ_1_| (the absolute difference between two means), σ is the variance of two means, n_1_ is the sample size for group 1, n_2_ is the sample size for group 2, α is the probability of type 1 error (usually .05), β is the probability of type 2 error (usually .2), and z is the critical *Z* value for a given α or β.

### Randomization and Allocation

A randomization chart has been generated with the help of computer-generated random numbers. Participants will be randomized in a ratio of 1:1 to either the study group or the control group. The randomization schedule will be strictly controlled and remain with the biostatistician/data analyst. Sequentially numbered, sealed, opaque envelopes will be used to conceal the allocation.

### Study Procedure

The participant will be screened for AR as per the inclusion criteria. Prior to any trial-related activity, the investigator will give the participants or their parents or guardians information about the trial verbally and in printed form. The investigator will ensure that the participant is fully informed about the aims, procedures, discomforts, and expected benefits of the trial. It must be emphasized that participation is voluntary, and participants have the right to discontinue the trial at any time without any prejudice.

**Figure 1:**
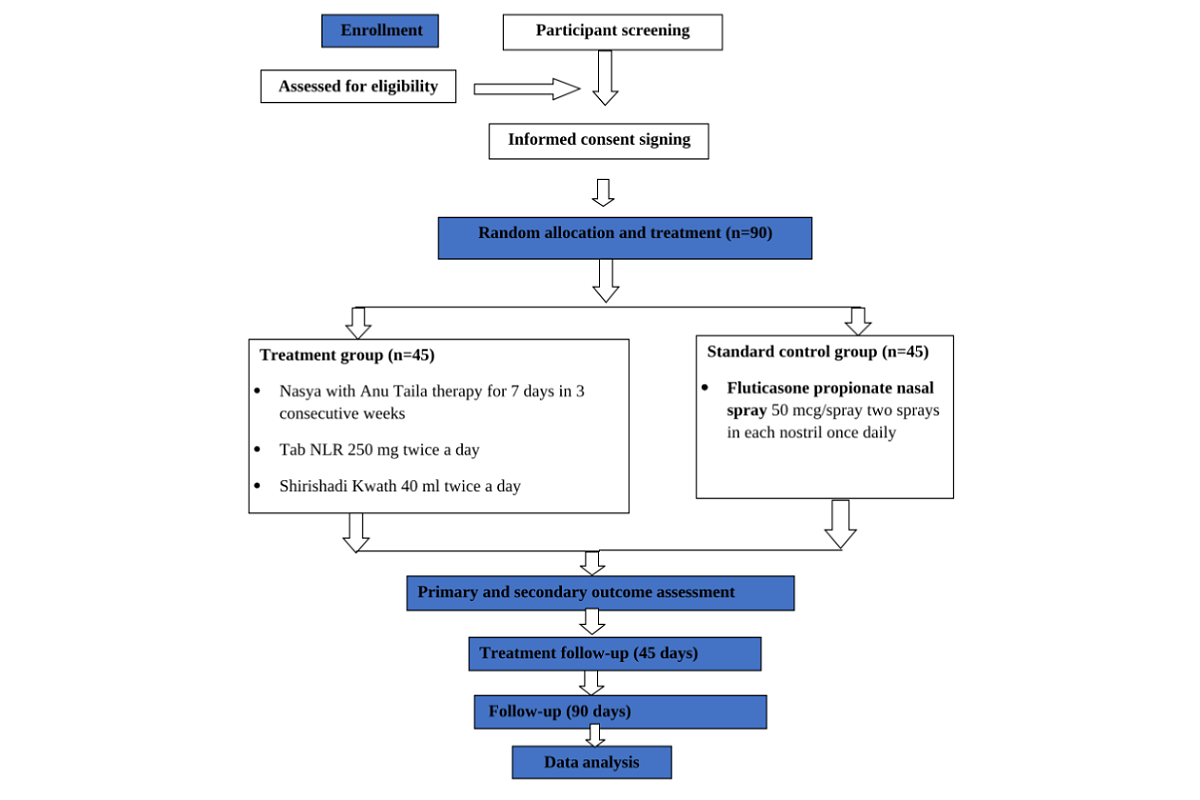
Study procedure. NLR: Naradiya Laxmivilas Rasa.

After the screening, suitable participants will be enrolled in the study after signing the consent form. The required tests will be carried out. Within 1 week or as soon as the laboratory results are received and found to be within the eligibility limits, the participant will be enrolled in the study, with this day being recorded as visit 1 or the baseline visit. The treatment will be given to her after fulfilling all the formalities as per the protocol. The subsequent follow-up will be on day 16 (visit 2), day 31 (visit 3), day 45 (visit 4), day 75 (visit 5), day 105 (visit 6), day 135 (visit 7), and completion of the active treatment (visit 8). Participants will visit the study site 8 times during the trial. The activity details during each visit are presented in Table 1 and Figure 1.

**Table 1:** Study schedule.

Component	Screening	Baseline	Days 16 and 31	Day 45	Days 75 and 105	Day 135
Informed consent	✓					
Demographics and medical history		✓				
Assessment of Prakriti		✓				
Laboratory tests	✓			✓		
Clinical examination		✓	✓	✓	✓	✓
Assessment of Ayurvedic parameters		✓		✓	✓	✓
Concomitant medication		✓	✓	✓		
Rescue medication			✓	✓		
Assessment of adverse drug reactions			✓	✓		
Assessment of drug compliance			✓	✓		
Issue of trial drugs		✓	✓			
Control of Allergic Rhinitis and Asthma Test score	✓			✓		
Diagnostic nasal endoscopy		✓		✓		✓

### Laboratory Tests

The laboratory tests (hematological/biochemical/ultrasound sonography) will be conducted at identified reputed laboratories or National Accreditation Board for Testing and Calibration Laboratories–accredited facilities. The laboratory tests and hematological examination carried out at baseline and the end of treatment (day 45) will collect the following values: hemoglobin (%), total leukocyte count, differential leukocyte count, erythrocyte sedimentation rate, absolute eosinophil count, fasting blood sugar, blood urea, serum creatinine, serum uric acid, total bilirubin, direct bilirubin, indirect bilirubin, serum alkaline phosphatase, aspartate aminotransferase, alanine aminotransferase, and total serum immunoglobulin E.

### Data Collection, Management, and Analysis

The data collection will include all information in the CRFs. All participants will be assigned an enrollment number, which will be used for CRFs and electronic databases. Consent forms and CRFs will be stored in locked cupboards, and electronic databases will be password protected. The data will be entered using the double-entry method for accuracy. All data will be accessible to the principal investigator and coinvestigators during and after the study, and will be available to sponsors and monitors as required. Data will be stored for 5 years and then destroyed. In case of dropout, 10% of the data will be inserted through the imputation technique. Proper documentation will be done to ensure accurate interpretation and verification related to the study. The analysis will be done by the CCRAS statistical unit. If any participants withdraw from the study prematurely, an attempt will be made to collect data for at least 1 month after enrollment, which includes the objective clinical and laboratory results. Data are important to the integrity of the final study analysis since early withdrawal could be related to the study drug’s safety profile.

### Statistical Methods

Continuous variables will be presented as means, SDs, medians, and ranges. Categorical variables will be summarized with frequencies and percentages. When inferential analyses are conducted for continuous variables, they will be tested for normality. Nonparametric methods will be used for comparison of nonnormal data. Within-group comparisons will be done by using paired *t* tests, while between-group comparisons will be done using independent sample *t* tests for the data with normal distribution. Nonnormal data within the group will be compared using the Wilcoxon sign rank test, while between-group comparisons will be done by using the Mann-Whitney *U* test. Categorical data between groups will be compared using the *χ*^2^ or Fisher exact test. A 2-sided *P* value <.05 will be considered statistically significant. Per-protocol and intention-to-treat analyses will be carried out at the end of the study. All data will be analyzed using STATA 16.1 (StataCorp).

### Ethical Considerations

This research protocol has been reviewed and approved by the Institutional Ethics Committee of Vardhman Mahavir Medical College, Safdarjung Hospital, New Delhi (IEC/VMMC/SJH/Project/2023-02/cc-320 dated 28.03.2023). Participants will receive an information sheet with the research details given in two languages (Hindi and English). Voluntary signed informed consent will be obtained from the participants before any clinical trial–related procedure. The participants will be informed by the investigator that all trial data recorded will be treated in strict confidence. During documentation and analysis of the trial, the individual participant will only be identified by their enrollment number.

Participant confidentiality will be guaranteed, and only researchers will have access to the data. Participants are nominally compensated for their loss of wages and conveyance charges by paying an amount of ₹100 (US $1.19) for every visit to the hospital during the study period. The safety of the trial intervention will be evaluated by recording the incidence of adverse events on every scheduled follow-up visit. All adverse events during the study timeline will be recorded and monitored per the International Council for Harmonisation of Technical Requirements for Pharmaceuticals for Human Use and Guideline for Good Clinical Practice guidelines. The investigator will report the same to the institutional ethics committee and the sponsors as soon as possible.

For facilitating appropriate reference standards for scientific, ethical, and safety issues before the trial begins, this protocol has been developed according to the 2013 SPIRIT (Standard Protocol Items: Recommendations for Interventional Trials) and CONSORT (Consolidated Standards of Reporting Trials) statements.

### Data Safety and Monitoring

After 25% of the participants complete the study, the data will be analyzed for the trial drugs’ safety. The project monitoring committee will monitor the trial’s progress through review meetings and site visits as per the requirement for ensuring strict adherence to the trial protocol and accurate completion of the CRFs.

### Drug Compliance

The compliance of taking trial drugs will be assessed at each visit during the follow-up visits (days 16, 31, 45, and 75) by assessing the approximate quantity consumed. The participants with ≥80% compliance will continue the study.

### Prior and Concomitant Medication

The participants will be instructed to not take medications other than the trial drugs for any disease without consulting the investigators. The investigator will instruct the participants to consult them for any other signs and symptoms or if anything unusual occurred. The investigator will record any other medications the participants took.

### Rescue Medication

Rescue medication will be used to alleviate any emergency condition at the investigator’s discretion and will be documented in detail in the CRF.

## Results

The project was funded by the CCRAS in February 2023. Participant recruitment was initiated on September 22, 2023. As of May 2024, we enrolled 72 participants in both groups. Data analysis will be completed by February 2025, and the results will be published afterward.

## Discussion

### Conclusions

Ayurveda scholars correlate AR with types of diseases (pratishyaya/pinasa; National Ayurveda Morbidity Code: I-1) based on the similarities in causes, clinical features, and treatment. This paper describes the protocol for the Ayurvedic management of AR. We undertook a randomized controlled trial to objectively assess the efficacy of Ayurvedic treatments for the management of AR and compared it with the gold standard intranasal corticosteroids for mild to moderate AR. We assessed the changes objectively by using the CARAT score and nasal endoscopy index as the outcomes for participants with AR.

The literature suggests that, based on the severity of the disease and condition of the patient, Ayurveda has multiple treatment modalities for this ailment, including Shodhana Karma (biocleansing therapy; Standard Ayurveda Terminology [SAT]: I.76) and Samshamana (palliative procedure; SAT: I.37) [23]. The primary treatment modality for managing diseases of the head and neck is Nasya (medication through the nasal route; SAT I.156). Most Ayurveda drugs and preparations used for the treatment of AR have been proven to have immunomodulatory, antiallergic, and anti-inflammatory properties [24]. Studies have demonstrated promising results of some Ayurveda interventions for managing AR. With this study, we aim to observe the effectiveness and safety of Ayurvedic management using the formulations of NLR, Shirishadi Kwath, and Anu Taila Nasya in reducing symptoms associated with AR, such as rhinorrhea, nasal itching, paroxysmal sneezing, nasal congestion, and obstruction. If beneficial, this study could contribute significantly to integrative approaches for managing AR.

### Strengths and Limitations

The strengths of this study include its randomized design and inclusion of enough participants to allow for adequate statistical power for subgroup analyses. This study uses the CARAT score and nasal endoscopy index, which are well-validated instruments to assess AR.

One potential limitation of this study is that the investigators were aware of the participants’ group assignment, and therefore, bias in favor of any particular group cannot be excluded. However, every effort was made to ensure blinding of the participants during the group allocation. An independent evaluator blind to study conditions was responsible for administering all study measures. A second limitation is the short intervention of the trial (ie, 45 days). However, the study follow-up was longer (ie, 90 days) to assess the recurrence of the symptoms/disease.

The results are expected to show better relief for AR for the treatment group. The protocol may also serve as a reference for the planning of similar clinical trials*.*

## Appendix

Multimedia Appendix 1 Control of Allergic Rhinitis and Asthma Test.

Multimedia Appendix 2 Nasal endoscopy index for patients with allergic rhinitis.

## Abbreviations

AR: allergic rhinitis
ARIA: Allergic Rhinitis and Its Impact on Asthma
CARAT: Control of Allergic Rhinitis and Asthma Test
CCRAS: Central Council for Research in Ayurvedic Sciences
CONSORT: Consolidated Standards of Reporting Trials
CRF: case report form
NLR: Naradiya Laxmivilas Rasa
SAT: Standard Ayurveda Terminology
SPIRIT: Standard Protocol Items: Recommendations for Interventional Trials

## Data Availability

The findings will be disseminated to the public through conference presentations and published in open-access peer-reviewed journals. Analyzed data will be made available for interested researchers with a reasonable request after the publication of the results.
