# Supplementary material for: Ayurveda Management of Allergic Rhinitis: Protocol for a Randomized Controlled Trial
Source: JMIR Res Protoc. 2024 Sep 25;13:e56063. doi: 10.2196/56063 (PMC11464932; doi:10.2196/56063)
Supplement: Multimedia Appendix 1 [file resprot_v13i1e56063_app1.docx]

**Appendix I**

**CARAT score (Control of Allergic Rhinitis and Asthma Test)**

Due to your allergic respiratory diseases (asthma, rhinitis, allergies) in the last four weeks, on average, how many times did you have:

| Sr.No. | Symptoms | Never | 1 or 2 days per week | More than 2 days per week | Almost every day |
| --- | --- | --- | --- | --- | --- |
| 1. | Blocked Nose? | 3 | 2 | 1 | 0 |
| 2. | Sneezing? | 3 | 2 | 1 | 0 |
| 3. | Itchy Nose? | 3 | 2 | 1 | 0 |
| 4. | Runny nose? | 3 | 2 | 1 | 0 |
| 5. | Shortness of breath/dyspnoea? | 3 | 2 | 1 | 0 |
| 6. | Wheezing in the chest? | 3 | 2 | 1 | 0 |
| 7. | Chest tightness upon physical exercise? | 3 | 2 | 1 | 0 |
| 8. | Tiredness/limitations in doing daily tasks because of your allergic respiratory diseases? | 3 | 2 | 1 | 0 |
| 9. | Woke up during the night because of your allergic respiratory diseases? | 3 | 2 | 1 | 0 |
| In the last 4 weeks, because of your allergic respiratory diseases, how many times did you have to increase the use of medication | | I am not taking any medicines | Never | Less than 7 days | 7 or more days |
| 10. | Increased the use (dosage or frequency) of your medicine? | 3 | 3 | 2 | 0 |

-------------Score

(Sum of all 10 questions, 0-worst, best -30)

Date--/--/----

---------------------------------------------------------------------------------------------------------------
