# Supplementary material for: Ayurveda Management of Allergic Rhinitis: Protocol for a Randomized Controlled Trial
Source: JMIR Res Protoc. 2024 Sep 25;13:e56063. doi: 10.2196/56063 (PMC11464932; doi:10.2196/56063)

**Appendix II**

**Diagnostic Nasal Endoscopy Index in patients with AR**

| **Parameters** | | **Score** | | |
| --- | --- | --- | --- | --- |
| **Nasal membrane color** | **Pale** | **0 (Normal)** | **1 (Mild)** | **2 (Severe)** |
|  | **Hyperemia** |  |  |  |
| **Rhinorrhea** | **Watery** |  |  |  |
|  | **Yellow** |  |  |  |
| **Inferior turbinate swelling** | **Hypertrophy** |  |  |  |
|  | **Atrophy** |  |  |  |


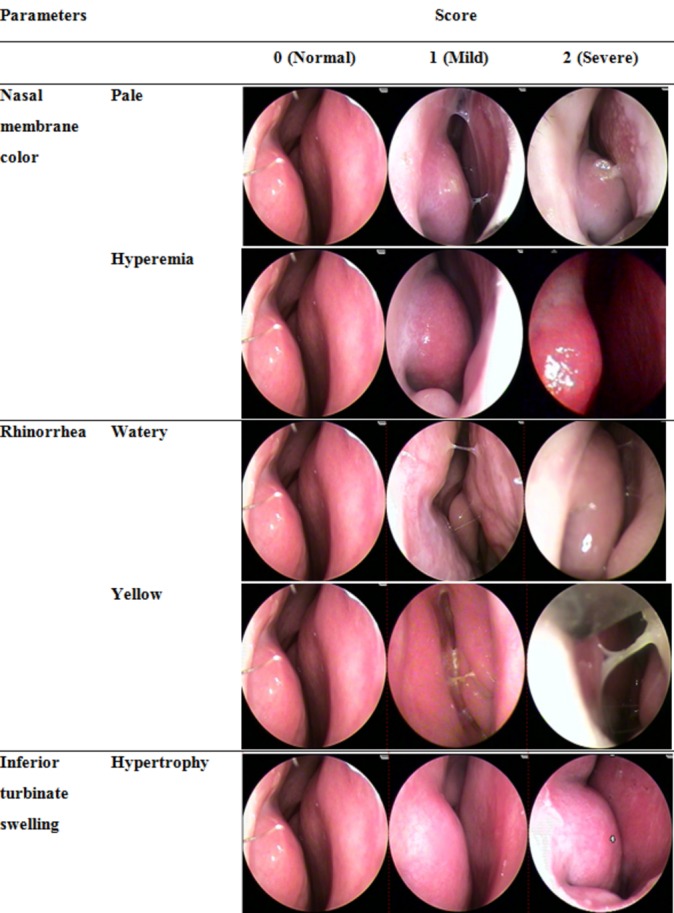

Supplement: Multimedia Appendix 2 [file resprot_v13i1e56063_app2.docx]
